# Study on Mechanical Properties of Two-Component Polyurethane Based on Multi-Scale Molecular Simulation

**DOI:** 10.3390/ma16031006

**Published:** 2023-01-21

**Authors:** Xingyu Wang, Tianlai Yu, Yuxuan Wu, Yingjie Sheng, Yifan Wang, Yutong Hang

**Affiliations:** 1 School of Civil Engineering, Northeast Forestry University, Harbin 150040, China; 2 School of Civil Engineering, Dalian University of Technology, Dalian 116081, China

**Keywords:** urethane elastomer, molecular dynamics simulation, mechanical property, temperature, isocyanate content

## Abstract

Mechanical properties determine the use of two-component polyurethane materials. The compatibility of two components in the polyether polyol-MDI molecular system greatly influences the formation of mechanical properties in polyurethane materials. In this paper, we studied and evaluated the compatibility and mechanical properties of two-component polyurethane at multiple scales by combining molecular dynamics simulation with macroscopic experiments, which is an important guideline for synthesizing and preparing two-component polyurethanes. We evaluated the stability of the two-component polyurethane system by calculating the solubility parameter, binding energy, and diffusion coefficient at four temperatures with three isocyanate contents. The Perl scripting language obtained the mechanical properties of the MDI-polyether polyol system. The MD calculation results show that the solubility parameter of two-component polyurethane negatively correlated with temperature, and the intermolecular binding energy and MDI diffusion coefficient positively correlated with temperature. When the mass ratio of polyether polyol to isocyanate was 1:0.6, the solubility parameter difference between the two was 1.43 (J/cm^3^)^1/2^, the intermolecular binding energy was 531.68 kcal/mol, and the two-component system was more stable. A macroscopic direct tensile test was employed to assess the polyurethane elastomers’ tensile properties. Our results show that the tensile strength of polyurethane elastomers increased with the increase in isocyanate content and decrease in temperature. Furthermore, the elongation at the break decreased, and the modulus increased, which is consistent with the law of molecular simulation.

## 1. Introduction

Polyurethane material is synthesized by the polycondensation of low-molecular-weight polyether/polyester and polyisocyanate. The main molecule chain comprises soft and hard segments. The different types and contents of two-component raw materials lead to differences in the number and structure of soft and hard chains, which causes the material to show various properties and mechanical characteristics [1,2]. Therefore, polyurethanes are polymers available for many technical applications requiring flexibility, durability, and impact resistance [3,4,5]. Studies showed that polyurethane elastomers synthesized by macromolecular MDI and polyether have good hydrolysis resistance and low temperature flexibility, and are widely used in the construction field [6,7].

Mechanical properties determine the use of polyurethane elastomers. However, due to the complex composition and reaction mechanism of two-component polyurethane, current research on the mechanical properties of polyurethane elastomers is mainly based on macroscopic experiments. Researchers often determine the effects of two-component content, additives, and the external environment on polyurethane elastomers’ mechanical properties by testing the mechanical properties of polyurethane elastomer specimens [8,9]. In addition, the two-component polyurethane reaction’s degree of polymerization is also an important factor affecting its mechanical properties. Polyurethane synthesis is mainly the reaction between isocyanates and active hydrides (hydroxyl) in polyether polyol. The nucleophilic center of active hydride collides with the electrophilic center of isocyanate, causing the polymerization of two additional molecules [10]. Therefore, the size of intermolecular interaction energy and the diffusion behavior of two-component molecules in the system affect the collision probability between molecules and the adequacy and rate of the polymerization reaction. However, studying the compatibility of two-component materials by macroscopic methods is difficult because the two-component polyurethane reacts very quickly after mixing. Molecular dynamics (MD) simulation technology provides a new way to solve this problem. It can accurately scale the time scale to the picosecond level and analyze the influence of component content and the external environment (temperature, pressure, etc.) on material properties at the nano-micro level [11,12].

Previous studies never predicted the mechanical properties of polyurethane materials in terms of the compatibility of the two-component blend. In addition, uniformly characterizing polyurethane is difficult on a molecular scale due to its complex composition. Performing molecular simulation calculations for two-component polyurethane is also difficult. Since polyether polyols and MDI are the main components of polyurethane elastomers, and the two are comparable on a nanoscale level, this paper selects the polyether polyol molecule–MDI molecular system as the research object. It uses Materials Studio molecular simulation technology and macroscopic experiments to study the two-component system at multiple scales. By analyzing the solubility parameters, binding energy, and molecular diffusion coefficient of the polyether polyol-MDI system at different temperatures and ratios, we judged the influence of temperature and ratio on the mixing effect of the two components. We used the Perl scripting language to calculate the mechanical properties of the two-component system and the relationship between stress and strain. Finally, we verified the microscopic mechanism obtained by molecular simulation using macroscopic experiments and analyzed the influence of temperature on the mechanical properties of two-component polyurethane molding specimens. These measures guide the formulation design of two-component polyurethane and the setting of each component parameter in the processing and production processes.

## 2. Simulation of Molecular Dynamics

### 2.1. Principles of Molecular Dynamics Simulation

The basic principle of MD theory is solving Newton’s equation of motion. First set the motion of all particles in the system by following Newton’s equation of motion. The calculation formula is

(1) $$\frac{\partial^{2}x_{i}}{\partial t^{2}}=\frac{Fx_{i}}{m_{i}}=a_{i}$$

(2) $$Fx_{i}=\frac{\partial E}{\partial x_{i}}$$

where in Formula (1), *t* represents time, and *x_i_*, *m_i_*, *a_i_*, and *F* represent the coordinates, mass, acceleration, and force of particles, respectively. In Formula (2), *x_i_* represents the coordinate of the particle, *E* represents the potential energy of the system, and the force *F* of the particle is equal to the first-order partial derivative of potential energy to the coordinate. MD simulation requires a rational selection of ensembles to reflect the actual physical process of materials. According to the different characteristics of the simulated object, the ensemble is divided into (1) micro-canonical ensemble (NVE); (2) canonical ensemble (NVT); (3) constant pressure, constant temperature (NPT); and (4) constant pressure, constant enthalpy (NPH). In the simulation of different ensembles, temperature and pressure balance control are usually needed to ensure that the simulation proceeds in the right direction. Equilibrium molecular dynamics calculates the molecular system optimization structure in different ensembles: energy, density, diffusion coefficient, heat capacity, etc. For the dynamic properties of the system, non-equilibrium molecular dynamics can be used to calculate the diffusion coefficient, thermal conductivity, viscosity, and other non-equilibrium properties of molecular systems.

### 2.2. Solubility Parameter

The solubility parameter (*δ*) is a physical index that predicts the compatibility between materials and is widely used in the field of polymer materials. Cohesive energy characterizes the cohesive properties of materials. Cohesive energy per unit volume is called cohesive energy density (CED). In polymers, the intermolecular force is a comprehensive reflection of various forces and cannot be expressed by a single force. Therefore, we used cohesive energy or cohesive energy density to measure the size of the force between macromolecules. In 1916, Hildebrand proposed the solubility parameter and defined it as the square root of the cohesive energy density of matter [13].

(3) $$\delta ={\left(E/V\right)}^{1/2}={\left(U_{m}/V_{m}\right)}^{1/2}$$

Here, *E* is cohesion energy (J/mol); *V* is the volume (mL/mol); *E* and *V* are the cumulative values of the energy and volume of the groups constituting the molecule; *U_m_* is the molar evaporation energy of the polymer; and *V_m_* is the molar volume of a repeating unit of the polymer.

### 2.3. The Energy of Binding

The essence of compatibility is the intermolecular interaction of each component. Molecular dynamics simulation accurately characterizes the strength of intramolecular and intermolecular interactions in the system. Molecular mechanics theory suggests that the total energy of the force field comprises intramolecular bonding interaction, intramolecular non-bonding interaction, and cross-form energy.

(4) $$E_{total}=E_{valance}+E_{non-bond}+E_{cross-term}$$

Here, *E_total_* is the total energy of the system; *E_valance_* is the bonding energy; *E_non-bond_* is a non-bonding energy; and *E_cross-term_* is the cross-interaction energy.

Intramolecular bonding interactions include bond stretching, bond angle bending, dihedral angle twisting, and off-plane bending. The role of intramolecular non-bonds includes Coulomb interaction and van der Waals force. Sometimes, intramolecular hydrogen bonds can also be used in intramolecular non-bond interactions. Binding energy (*E_bind_*) cannot be used as a sufficient criterion for blends’ compatibility; however, different binding energies between the blending systems can predict compatibility. The greater the binding energy, the more stable the formed group and the better the compatibility between polyether polyol and isocyanate. Therefore, the size of *E_bind_* is usually used to predict and compare the compatibility of similar systems. The binding energy (*E_bind_*) is the negative value of the interaction energy (*E_inter_*), i.e., *E_bind_* = −*E_inter_*.

(5) $$E_{bind}=-E_{\mathrm{inter}}=(E_{po}+E_{MDI})-E_{Total}$$

Here, *E_bind_* and *E_inter_* are the binding and interaction energies between the MDI-polyether polyol interface, respectively. *E_total_*, *E_po_*, and *E_MDI_* are the average single-point energies of the two-component system, polyether polyol, and the MDI molecule, respectively.

### 2.4. MSD and Diffusion Coefficient

According to Brown’s law, molecules are always moving irregularly, and the mean square displacement (MSD) can characterize the activity of molecular chains in the model or the diffusion activity of particles in the system. The higher slope or shift in the MSD curve usually indicates higher molecular mobility, which benefits the effective motion of the two-component molecules. The mean square displacement represents the statistical square of displaced particles in the system relative to the initial time within a period of time. The formula is

(6) $$\mathrm{MSD}=\langle {\left|r(t)-r(0)\right|}^{2}\rangle$$

where *r*(0) and *r*(*t*) are the atomistic positions of the center of mass at initial time 0 and later time *t*, respectively. The part in angular brackets denotes the mean square displacement (MSD) of all atoms.

In order to quantitatively compare the diffusion ability of MDI molecules to different contents, the slope of the curve versus time is obtained by fitting the MSD curve, which is substituted into the Einstein relation for calculating the molecular diffusion coefficient, and the diffusion coefficient *D* can be obtained [14]:

(7) $$D=\underset{x\to \infty }{\lim}\frac{\langle \left|r_{t}-r_{0}\right|\rangle }{6t}=\frac{s(t)}{6t}=\frac{k}{6}$$

where *D* is diffusion coefficient; *t* is time; *r*(*t*) is the coordinate of the molecular; and *k* is the slope of the MSD curve.

### 2.5. Mechanical Property

Mechanical properties are important properties in composite materials. In a molecular simulation, stress is generated inside the system under an external force so that the relative position of the particles changes. Only two independent coefficients (*λ* and *μ*) for isotropic materials are needed to fully describe their stress–strain behavior.

(8) $$E=\frac{\mu (3\lambda +2\mu )}{\lambda +\mu }\;\;K=\lambda +\frac{2}{3}\mu \;\;G=\mu$$

Here, *λ* and *μ* are Lamé constants; *E* is the Young’s modulus of the material; *K* is the bulk modulus of the material; and *G* is the shear modulus of the material.

### 2.6. Construction of the Molecular Models

**Figure 1:**
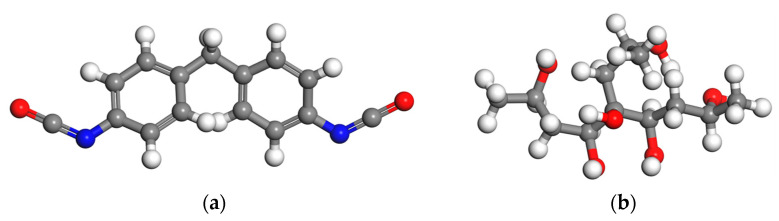
Molecular model of two-component materials. (**a**) MDI; (**b**) polyether polyol molecule (white represents the H atom, gray represents the C atom, red represents the O atom, and blue represents the N atom).

The molecular models of isocyanate and polyol are shown in Figure 1, where isocyanate is MDI.

**Figure 2:**
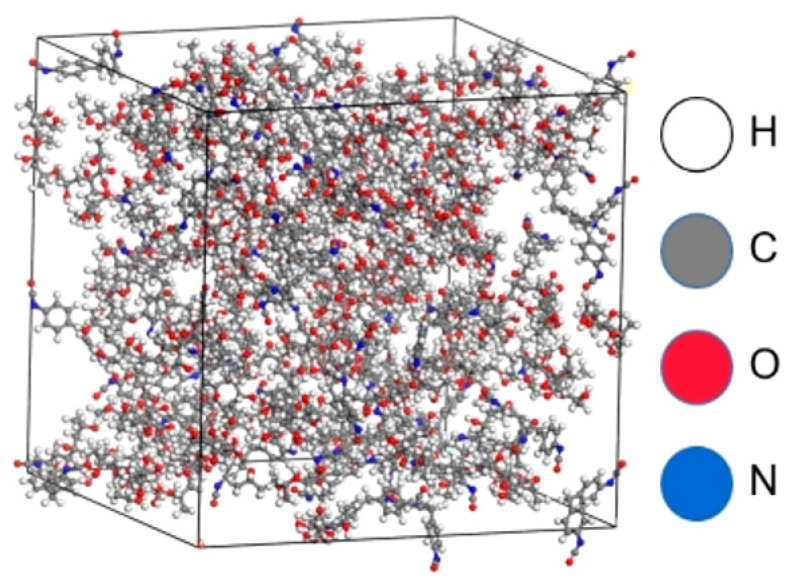
Composite model of ether polyol and isocyanate.

We present three kinds of polyurethane research ratios in this paper. The mass ratios of polyether polyol (component A) to MDI (component B) were 1:0.5, 1:0.55, and 1:0.6, respectively. Then, we converted the mass ratio to the molar ratio in the amorphous cell section of MS software for two-component model construction. Under the COMPASS [15] force field, we selected the geometry optimization function in the Forcite module to perform a 100,000-step geometric optimization of the established model. Subsequently, the anneal function in the Forcite module was selected to anneal the model. We performed the temperature rise between 298.15 and 798.15 K. The temperature drop was performed between 798.15 and 298.15 K, and the dynamics module was used for 100 ps dynamic operation. At this time, the model reached a stable state. The stable composite model is shown in Figure 2. In the diagram, white represents H atoms, gray represents C atoms, red represents O atoms, and blue represents N atoms.

**Figure 3:**
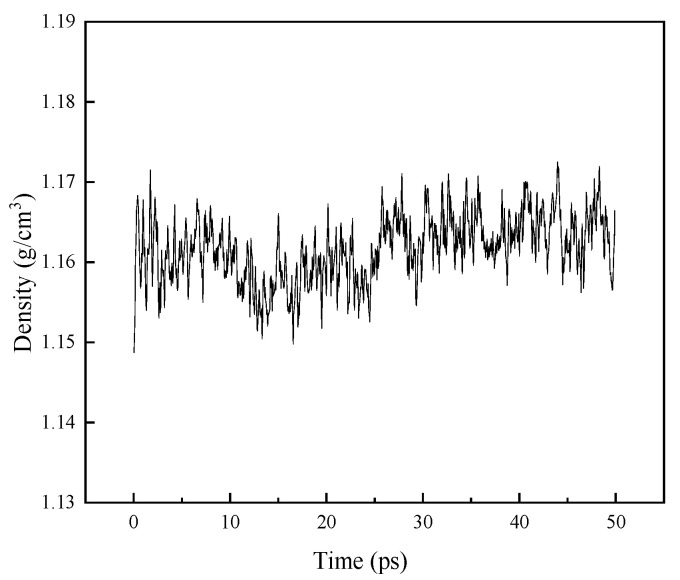
Density curve of two-component model.

Figure 3 shows the density of the model after the dynamic operation. The model density is around 1.16 g/cm^3^, which is close to the real density of the material, indicating that the model built for this paper is close to the real state and is highly credible [16].

## 3. Materials and Methods

### 3.1. Materials

We conducted laboratory tests to evaluate the performance of polyurethane elastomer raw materials. The physical and chemical properties of each component are shown in Table 1.

### 3.2. Specimen Preparation

We prepared three kinds of polyurethane elastomers by tensile testing. The two-component ratio is shown in Table 2, named PB1, PB2, and PB3. Isocyanates’ ability to produce a foaming reaction with water may lead to the formation of holes in the interior and surface of the specimen, thus affecting its performance. Therefore, before the two-component mixing, the polyether polyol should be sampled and tested to ensure that the sample’s mass fraction of water is <0.05%; then, the isocyanate is added and stirred at 1500 rpm for 3 min.

### 3.3. Mechanical Test

**Figure 4:**
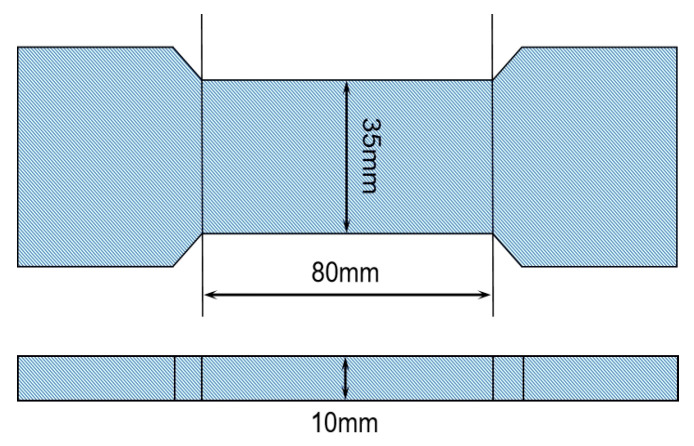
Dumbbell sample.

The polyurethane elastomer tensile specimen size is shown in Figure 4. We conducted 12 groups of tensile tests at three ratios and four temperatures. The temperatures used in the tests were 60, 20, −15, and −30 °C, and each group was tested three times. We conducted the tensile test on the Changchun Kexin DWD-100 universal testing machine. During the tensile test, we aligned the specimen’s center line with the center of the testing machine’s fixture, stretched the specimen at a tensile rate of 5 mm/min until it broke, and recorded the load and displacement. We stored the test piece in a low-temperature environment for 6 h prior to low-temperature working conditions. The tensile strength and elongation at break were calculated according to Formulas (9) and (10), respectively.

(9) $$\delta =\frac{P}{A}$$

Here, *δ* denotes the tensile strength of the polyurethane elastomer specimens (MPa); *P* is the ultimate load (N); and *A* is the initial cross-sectional area (mm^2^).

(10) $$L=\frac{L_{1}-L_{0}}{L_{0}}\times 100\%$$

Here, *L* denotes the elongation at the break of the polyurethane elastomer test piece (%); *L*_1_ represents the distance between the marks when the specimen is broken (mm); and *L*_0_ denotes the distance between the front marks of the stretch (mm).

## 4. Multi-Scale Molecular Simulation Results and Analysis

### 4.1. Solubility Parameter Analysis

The solubility parameter can be used as an index to judge the compatibility of two-component mixing (the smaller the difference in solubility parameters, the better the compatibility). Table 3 shows the solubility parameters of the molecular model at different two-component ratios and temperatures.

As shown in Table 3, the solubility parameters of the two components decrease with the increase in temperature due to the high temperature, which increases the kinetic energy and macroscopic volume of the polymer molecules, intensifying the thermal motion and resulting in a gradual decrease in the molecules’ cohesive energy density and solubility.

**Figure 5:**
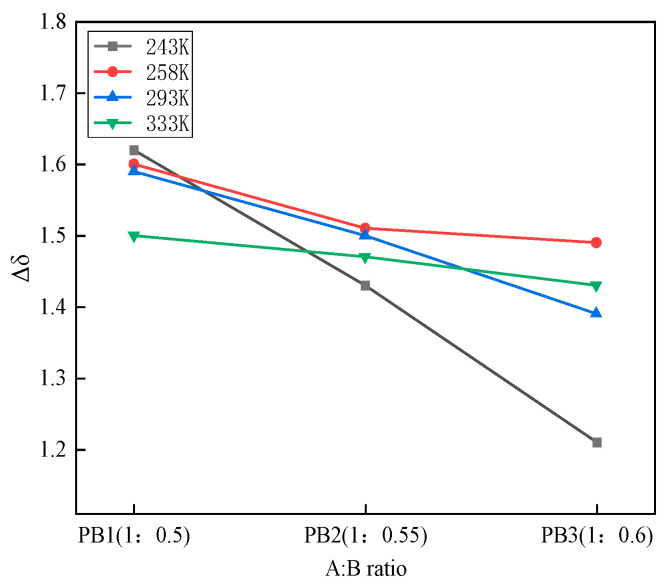
Difference of solubility parameters.

According to the study, when the difference of solubility between polyol and MDI is less than two, the two can be compatible [17]. As shown in Figure 5, the difference in solubility parameters (Δ*δ*) between the two molecular models at different temperatures was generally minor, ranging from 1.2 to 1.7 (J/cm^3^)^1/2^. At 333 K, the two-component solubility parameter difference satisfies Δ*δ* < 1.4–1.5 (J/cm^3^)^1/2^, indicating that the molecular system is the most stable. When the mass ratio of polyether polyol to isocyanate is 1:0.6, the solubility parameter difference between the two components is smaller than other ratios, indicating that the two-component system is more stable with increased isocyanate content. However, the difference in solubility parameters of the same two-component ratio at different temperatures did not show a certain rule. This finding is due to the different sizes and structures of polyether polyols and MDI molecules, resulting in different decrease rates of cohesive energy density and solubility parameters with temperature. It is also related to the physical properties of polyether polyols and MDI.

### 4.2. Binding Energy Analysis

**Figure 6:**
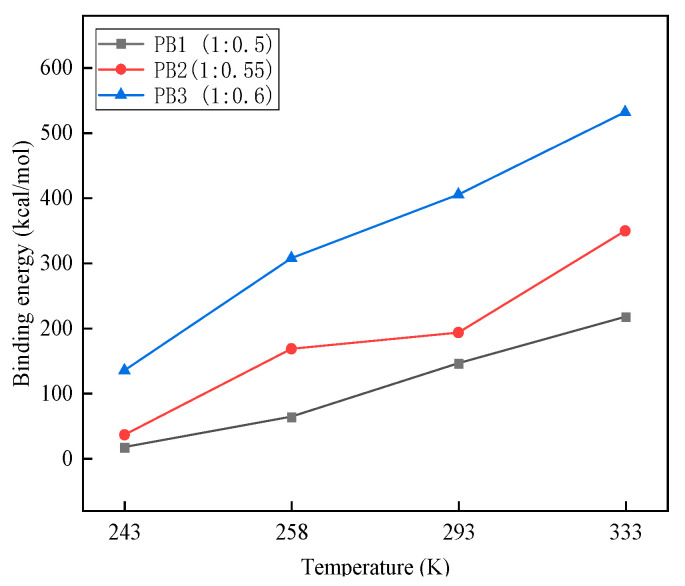
The binding energy of each ratio at different temperatures.

We calculated the total energy averages for the last 10 frames of the steady-state structures of the three two-component models with mass ratios of 1:0.5, 1:0.55, and 1:0.6 at four temperatures of 243, 258, 293, and 333 K using molecular dynamics. The results are shown in Table 4. The results of the binding energy calculations are shown in Figure 6.

The greater the intermolecular interaction energy of the system, the stronger its intermolecular interaction ability and stability. When the interaction energy is positive, the molecules are mutually exclusive. When the interaction energy is negative, the molecules are attractive [18]. We mainly studied the interaction between polyether polyols and MDI molecules. The total potential energy primarily comprises intermolecular van der Waals and electrostatic energies, as shown in Figure 6. The binding energy of polyether polyols and MDI molecules increases with the decrease in the A:B ratio and increase in temperature. This finding indicates that the two-component molecular system is more stable with the increase in isocyanate content and better compatibility between composite materials.

### 4.3. MSD and Diffusion Coefficient Analysis

**Figure 7:**
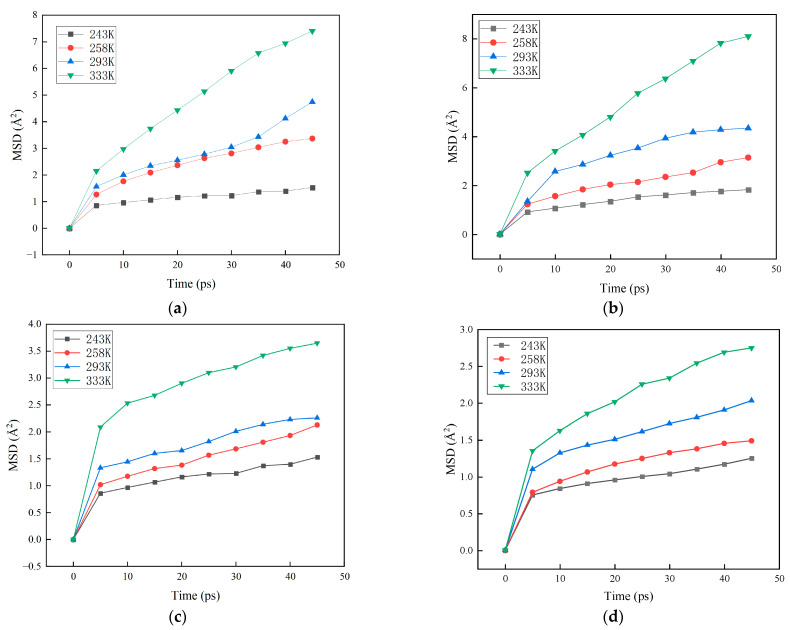
MSD curves of components at different ratios. (**a**) 0.5 MDI; (**b**) 0.55 MDI; (**c**) 0.6 MDI; and (**d**) polyether polyol.

We obtained the mean square displacement curves of the two components at different ratios using molecular dynamics simulation of the two-component miscible system. The results are shown in Figure 7.

The diagram shows that the MSD values of each component under different proportions increase with simulation time. The MSD curve is approximately linear after stabilizing. The higher the temperature, the greater the value of MSD. The MSD value of isocyanate is greater than that of polyether polyol. We analyzed MSD images and found that the two-component MSD image was not a straight line. The curve’s trend is steep initially and tends to flatten at the latter position. Therefore, a straight line was selected as the research object when calculating the slope; moreover, the MSD of the linear interval was selected to calculate the diffusion coefficient. We repeatedly adjusted the range of values to find the most reasonable value.

The diffusion coefficients of MDI and polyether polyol molecules at different two-component ratios are shown in Table 5, which intuitively reflect the kinematic ability of the two-component blend system.

Table 5 shows that the diffusion coefficient of molecules in the system increases with the increase in temperature, which two aspects can explain. Firstly, the increase in temperature enhances each molecule’s movement. Secondly, with the increase in temperature, the polyurethane elastomer heats and expands, the spacing between molecules increases, and the free volume inside the material increases. The holes for molecules to jump through increase [19], increasing the probability of molecules jumping between holes.

In addition, with the increase in isocyanate content, the diffusion rate of MDI molecules gradually slowed down. When A:B = 1:0.6, the diffusion coefficient of the MDI molecule was the smallest because as the number of MDI molecules increases, the density of molecules in the system increases, and the free volume inside the material decreases, decreasing the jump probability of MDI molecules. On the other hand, with the increase in MDI molecular content, the interaction between MDI molecules is enhanced, which also reduces the jump diffusion of MDI between pores in the system.

### 4.4. Mechanical Performance Analysis

When using MD to calculate the mechanical properties of the system, the isocyanate-polyether-polyol molecular model is subjected to stress in all directions (six directions). The modulus also changes as the relative position of the particles in the system changes. When simulating the mechanical properties of the isocyanate-polyether polyol system, we set the simulation temperatures to 243, 258, 293, and 333 K. The modulus of each system was calculated by Formula (8). The MD simulation results are shown in Table 6.

**Figure 8:**
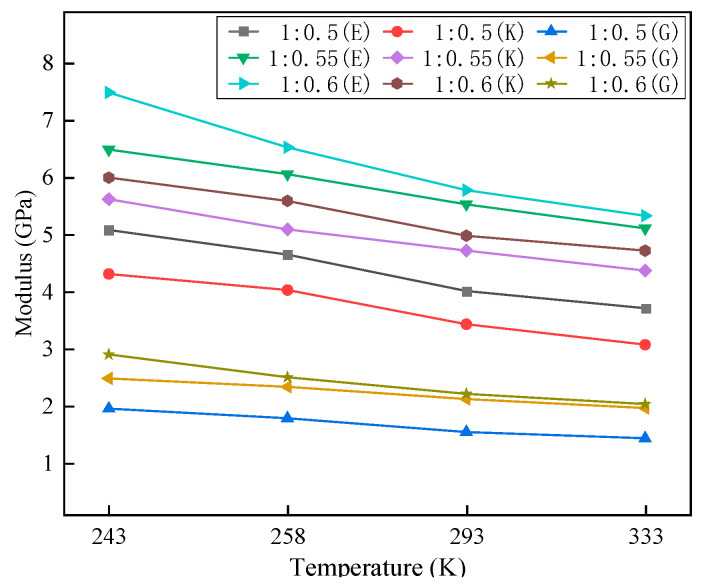
This physical modulus of two-component materials.

The modulus of the two-component molecular model is shown in Figure 8, and the simulation results are in the same order of magnitude as Young’s modulus of polyurethane elastomers with similar structures in the literature [12]. As the temperature increases, *E*, *K*, and *G* decrease because, under the stimulation of thermal energy, when the molecule is disturbed, the vibration of the atoms in the system strengthens, and the atoms move in a large range. The slip between molecules increases, resulting in a decrease in modulus. However, as the isocyanate content increases, its mechanical properties significantly improve regardless of the temperature.

## 5. Results and Analysis of Direct Tensile Tests

**Figure 9:**
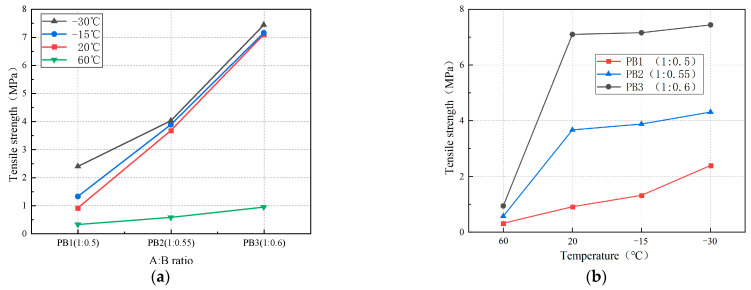
Effect on tensile strength. (**a**) Effect of the A:B ratio on tensile strength; **(b**) effect of temperature on tensile strength.

Figure 9 shows the effects of the A:B ratio and temperature on the tensile strength of polyurethane elastomers. As shown in Figure 9a, the elastomers’ tensile strength increased significantly with the A:B ratio decrease. At 20, −15, and −30 °C, the tensile strength of PB3 was 7.8, 5.42, and 3.11 times that of PB1, respectively. At 60 °C, the tensile strength of polyurethane elastomers increased slowly with the decrease in the A:B ratio. The material’s tensile strength was <1 MPa because polyurethane elastomers have difficulty releasing internal heat at high temperatures, resulting in a decreased degree of physical cross-linking of chain segments. Therefore, it is difficult to demonstrate high tensile strength [20]. Figure 9b shows that the polyurethane elastomer’s tensile strength increases with the temperature decrease. When the temperature is <20 °C, the growth rate of the elastomer’s tensile strength increases gradually. The tensile strengths of PB1, PB2, and PB3 increased by 45.1, 5.7, and 0.8%, respectively, when the temperature decreased from 20 to −15 °C. The tensile strengths of PB1, PB2, and PB3 increased by 81.1, 11.1, and 3.9%, respectively, when the temperature decreased from −15 to −30 °C because the crystallization rate between the molecules inside the polyurethane gradually increased as the temperature decreased, resulting in difficult movement between the segments. Therefore, the tensile strength of the elastomer gradually increases.

**Figure 10:**
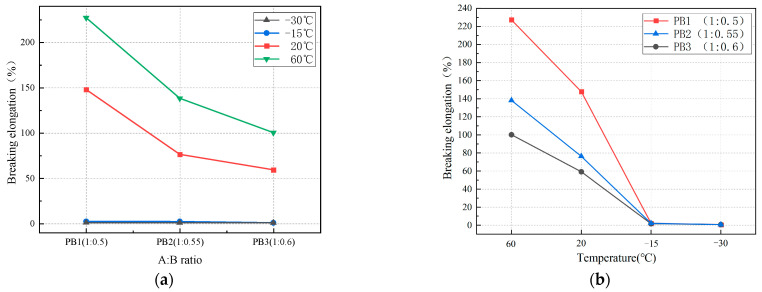
Effect on elongation at the break. (**a**) Effect of the A:B ratio on elongation at the break; (**b**) effect of temperature on elongation at the break.

**Figure 11:**
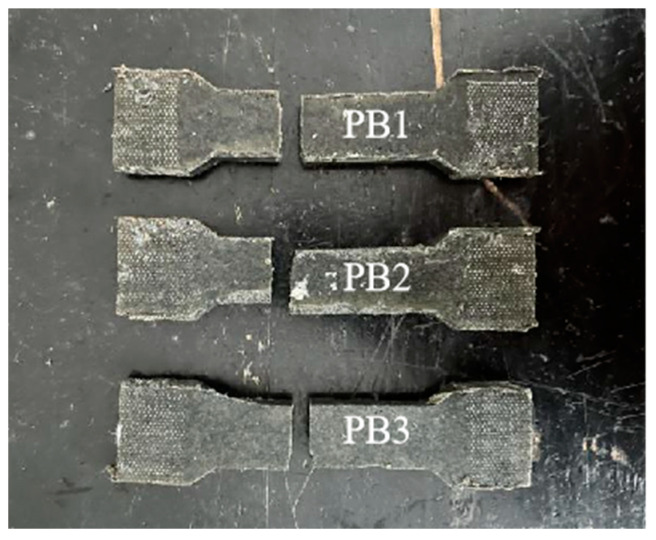
Failure mode of tensile specimen at −15 °C.

Figure 10 shows the effects of the A:B ratio and temperature on the elongation at the break of polyurethane elastomers. Figure 10a shows that the elongation at the break of polyurethane elastomers decreases with the decrease in the A:B ratio. At 60 and 20 °C, the elongation at the break of PB3 decreased by 127.1 and 88.72%, respectively, compared to PB1. Figure 10b shows that the elongation at the break of polyurethane elastomers decreases with the decrease in temperature. When the temperature decreased from 60 to −15 °C, the elongation at the break of PB1, PB2, and PB3 decreased by 225, 136, and 98.6%, respectively. At −15 °C, with the increased tension, the specimen had no obvious deformation. When the tension reached a certain value, the specimen suddenly broke, and the failure section was neat (Figure 11). When we reduced the temperature to −15 °C, the properties of the elastomer changed, and the material exhibited brittle characteristics. At this time, the elongation at the break of the elastomer was minor. The elongation at the breaks of PB1, PB2, and PB3 were 2.13, 2.04, and 1.63%, respectively. When the temperature decreased from −15 to −30 °C, the elongation at the break of the elastomer decreased slowly and tended to the horizontal line. At this time, the elongation at the break of the elastomer was about 0.6%.

**Figure 12:**
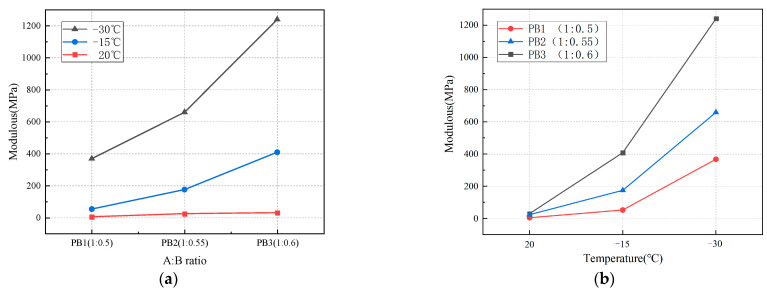
The influence of Young’s modulus. (**a**) Effect of the A:B ratio on Young’s modulus; (**b**) effect of temperature on Young’s modulus.

Figure 12 shows the influence of the A:B ratio and temperature on Young’s modulus of polyurethane elastomers. As shown in Figure 12a, Young’s modulus of polyurethane elastomers continues to increase as the A:B ratio decreases. Young’s modulus of elastomer increased more significantly with the increase in isocyanate content at low temperatures than at room temperature. At 20, −15, and −30 °C, Young’s modulus of PB3 increased by 25.4, 356, and 872.3 MPa, respectively, compared with PB1. As shown in Figure 12b, Young’s modulus of polyurethane elastomers continues to increase as the temperature decreases. When the temperature decreased from 20 to −30 °C, Young’s modulus of PB1, PB2, and PB3 increased by 363.2, 635.4, and 1210.1 MPa, respectively. The decrease in temperature and A:B ratio enhances the polyurethane elastomers’ ability to resist deformation.

In summary, the polyurethane elastomer’s tensile strength and Young’s modulus increased with increased isocyanate content, whereas the elongation at the break decreased. This result is mainly because the hard segments of polyurethane elastomers are mainly provided by isocyanate. With the increase in hard segment content, the hard segments are connected, and the number of hydrogen bonds increases, leading to increased interaction and physical cross-linking between the segments [21]. Therefore, the elastomer exhibits a higher Young’s modulus and mechanical strength at the macro level. However, increases in the degree of physical cross-linking between the segments hinder the movement of the macromolecular chain of the elastomer material. Thereby, the deformation of the elastomer material is inhibited, and the elongation at the break decreases. In addition, the soft segment can provide certain flexibility and elasticity to the elastomer. However, due to the increase in hard segment content, the proportion of soft segment gradually decreases, and the soft segment’s effect gradually weakens, which is also a reason for the decrease in elongation at the break of the elastomer.

In addition, with the decrease in temperature, the tensile strength and modulus of polyurethane elastomers increased, and the elongation at break decreased. This result is because the glass transition temperature of polyurethane elastomers is often lower than room temperature [22]. The decrease in temperature increases the crystallinity of polyurethane elastomers and makes the chain segments difficult to move. Therefore, the elastomer exhibits a brittle-hard state macroscopically. This finding is consistent with Young’s modulus of polyurethane elastomers, which increase with the increase in isocyanate content and the decrease in temperature.

## 6. Conclusions

1. The solubility parameters of polyether polyol and MDI decrease with the increase in temperature. When the temperature is 333 K, the solubility parameter difference between the two components is from 1.4 to 1.5 (J/cm^3^)^1/2^, and they blend best. When the mass ratio of polyether polyol and MDI is 1:0.6, the solubility parameter difference between the two components is minor compared with other ratios, indicating that the two-component system is more stable with increased isocyanate content.
2. The increasing MDI content and temperature increase the binding energy between two-component molecules. When the temperature is 333 K, and the mass ratio of the two components is 1:0.6, the maximum value is 531.68 kcal/mol, and the stability of polyether polyol and the MDI system is optimal.
3. As the temperature increases, the molecular motion in the system accelerates, and the diffusion coefficient continues to grow. The diffusion coefficient of MDI molecules decreases with the increase in MDI molecular content.
4. The macroscopic tensile test results show that the isocyanate content and temperature change significantly affected the mechanical properties of polyurethane elastomers. With the increase in isocyanate content and the decrease in temperature, the tensile strength of polyurethane elastomers increased, the elongation at break decreased, and Young’s modulus increased, which is consistent with the law of molecular simulation.

## Figures and Tables

**Table 1:** Physical and chemical properties of polyurethane components.

Polyurethane Components	Serial Number	Index	Specifications
Isocyanate	1	Appearance	Brown liquid
	2	Viscosity (25 °C)/mPa·s	175–240
	3	Isocyanate content/%	29.5~31.0
Polyether polyol	1	Appearance	Colorless to light yellow transparent liquid
	2	Viscosity(25 °C)/mPa·s	300–500
	3	Hydroxyl value/(mg KOH/g)	450–460
	4	Density (25 °C)/(g/cm^3^)	1.12 ± 0.20
	5	Acid value (mg KOH/g)	≤0.07
	6	Moisture content/%	≤0.05
	7	pH value	5.0–8.0

**Table 2:** Two-component ratio of polyurethane elastomer.

Code	PB1	PB2	PB3
A:B Ratio	1:0.5	1:0.55	1:0.6

**Table 3:** Solubility parameter (J/cm^3^)^1/2^.

Temperature (K)	A (1)	B (0.6)	B (0.55)	B (0.5)
243	24.56	23.35	23.13	22.94
258	24.38	22.89	22.87	22.78
293	24.07	22.68	22.57	22.48
333	23.59	22.16	22.12	22.09

**Table 4:** The energy of each system at different temperatures (kcal/mol).

Temperature (K)	System Energy						
	*E_T_* (0.5)	*E_T_* (0.55)	*E_T_* (0.6)	*E_po_*	*E_MDI_* (0.5)	*E_MDI_* (0.55)	*E_MDI_* (0.6)
243	−10,063.88	−10,291.62	−10,437.17	−7805.81	−2274.97	−2521.61	−2766.08
258	−9767.51	−9839.67	−9983.38	−7652.49	−2178.57	−2355.16	−2637.89
293	−8911.02	−9037.28	−9076.80	−7034.68	−2022.17	−2195.6	−2447.17
333	−8159.07	−8155.86	−8230.73	−6557.89	−1818.47	−1947.26	−2204.52

**Table 5:** The diffusion coefficient of polyether polyol molecules and MDI molecules.

Temperature (K)	A (1)		B (0.5)		B (0.55)		B (0.6)	
	D	R^2^	D	R^2^	D	R^2^	D	R^2^
243	2.09 × 10^−12^	0.9851	5.64 × 10^−12^	0.9627	2.46 × 10^−12^	0.9897	1.65 × 10^−12^	0.9339
258	2.31 × 10^−12^	0.9866	6.40 × 10^−12^	0.9905	3.67 × 10^−12^	0.9760	2.56 × 10^−12^	0.9883
293	3.42 × 10^−12^	0.9954	1.67 × 10^−11^	0.9679	5.27 × 10^−12^	0.9530	3.64 × 10^−12^	0.9281
333	4.46 × 10^−12^	0.9707	1.86 × 10^−11^	0.9781	9.35 × 10^−12^	0.9661	4.49 × 10^−12^	0.1338

**Table 6:** Calculation results of mechanical properties.

Temperature (K)	Index					
	A:B	*λ*	*μ*	*E* (GPa)	*K* (GPa)	*G* (GPa)
243	1:0.5	3.01	1.95	5.08	4.31	1.95
	1:0.55	3.97	2.48	6.49	5.62	2.48
	1:0.6	4.07	2.9	7.49	6	2.9
258	1:0.5	2.84	1.78	4.65	4.03	1.78
	1:0.55	3.54	2.33	6.06	5.09	2.33
	1:0.6	3.93	2.5	6.53	5.59	2.5
293	1:0.5	2.4	1.54	4.01	3.43	1.54
	1:0.55	3.31	2.12	5.53	4.72	2.12
	1:0.6	3.51	2.21	5.78	4.98	2.21
333	1:0.5	2.12	1.43	3.71	3.07	1.43
	1:0.55	3.06	1.96	5.11	4.37	1.96
	1:0.6	3.37	2.03	5.33	4.72	2.03

## Data Availability

The data used to support the findings of this study are available from the corresponding author upon request.
